# Immunohistochemical Overexpression of Cyclin D1 in Tunisian Invasive Breast Carcinoma Women

**DOI:** 10.34172/aim.2022.41

**Published:** 2022-04-01

**Authors:** Lobna Bouzidi, Saadia Makni, Jihen Feki, Rim Kallel, Soumaya Graja, Naourez Gouiaa, Tahya Sellami-Boudawara, Manel Mellouli

**Affiliations:** ^1^Department of Pathology and Research laboratory LR18SP10, University Hospital Habib Bourguiba, Sfax, Tunisia; ^2^Medical School of Sfax, University of Sfax, Sfax, Tunisia; ^3^Department of Medical Oncology, University Hospital Habib Bourguiba, Sfax, Tunisia

**Keywords:** Breast cancer, Cyclin D1, Estrogen receptor, Progesterone receptor, Prognosis

## Abstract

**Background:**

Breast cancer represents the most frequent cancer and cause of death in women worldwide and in Tunisia. Cyclin D1 is a gene of cell cycle regulation. It represents a potential oncogene in invasive breast cancer; however; the results are conflicting. We performed a retrospective study aiming to analyze the prognostic impact of cyclin D1 expression in patients with invasive breast carcinoma of no special type and its relation with clinical-pathological features.

**Methods:**

One hundred cases of invasive breast carcinoma of no special type diagnosed between 2009 and 2011 were included in this study. Immunohistochemical (IHC) staining was performed for cyclin D1 in all cases. Results were analyzed statistically.

**Results:**

Cyclin D1 positivity was seen in 74 cases (74%), of which 32 cases (32%) showed strong immunoreactivity. Cyclin D1 staining was statistically significantly associated with estrogen receptor (ER) and progesterone receptor (PR) positivity (*P*<0.0001) and with low grade SBR (*P*=0.007). None of the clinical data and other pathological features had any association with cyclin D1 expression (*P*>0.05). Univariate analysis revealed that expression of cyclin D1 was not statistically associated with overall survival (OS) and disease-free survival (DFS) (*P*=0.459 and *P*=0.564, respectively).

**Conclusion:**

These results confirm that cyclin D1 overexpression can be employed as a beneficial prognostic marker and suggest that anti-cyclin D1 therapy may be efficient, especially for ER positive tumors.

## Introduction

 Breast cancer represents the most frequent cancer and cause of death in women worldwide and in Tunisia. Its mortality rate is 6.6%.^1^ This cancer represents a heterogeneous disease which includes several pathological and molecular subtypes showing different and unpredictable outcomes and treatment responses.^2^

 Classical and well-established clinical and pathological prognostic markers like progesterone receptor (PR), estrogen receptor (ER) and human epidermal growth factor 2 (HER2) are necessary and useful but they do not always predict the outcomes of these tumors.^3-5^

 The provision of new prognostic factors is crucial for better understanding of the molecular mechanism of carcinogenesis, which allows improving the outcome and supplying new therapeutic perspectives.^6^

 Several recent studies addressing cyclin D1 have shown the role of this protein in the development of a substantial proportion of tumors, including breast cancer, as well as the therapeutic management.^7,8^

 Cyclin D1 plays a dual role as one of the main proteins of cell cycle regulation and as a transcriptional factor.^9^ It is encoded by the gene, oncogenic c-terminal cyclin D1 (*CCND1*) or parathyroid neoplasia gene (*PRAD1*), located on chromosome 11q13.^9^ The deletion of cyclin D1 causes poor mammary gland development and delivered protection from the development of breast cancer.^10^ On the other hand, cyclin D1 overexpression leads to excessive mammary proliferation and thus, high incidence of BC.^11^ Indeed, it allows progression through G1-S phase by binding to cyclin-dependent kinase 4 (CDK4) and cyclin-dependent kinase 6 (CDK6).^12^

 However, the role of cyclin D1 overexpression in the pathogenesis and prognosis of breast cancer remains controversial; researchers have reported inconsistent and conflicting results.^6^

 The goals of our study were to estimate the expression of cyclin D1 in invasive breast carcinoma, to evaluate the relation of this expression with other clinical-pathological prognostic factors and to judge the prognostic significance of cyclin D1 expression.

## Materials and Methods

###  Clinical and Pathological Data

 This is a retrospective study of female patients presenting with breast carcinoma between 2009 and 2011. Patients originated from southern Tunisia. Surgical resection was performed for all patients at the Department of Gynecology and Obstetrics of the Hedi Chaker University Hospital (Sfax, Tunisia). The specimens (n = 100) were formalin-fixed and paraffin-embedded at the Department of Pathology of the Habib Bourguiba University Hospital (Sfax, Tunisia). Patients who received radiation before surgical resection or neoadjuvant chemotherapy were not included. We used Microsoft Excel version 7.0 and medical files archived at the department of Pathology of the Habib Bourguiba University Hospital (Sfax, Tunisia) to collect pathological data. Hematoxylin and eosin slides were also retrieved from the department of Pathology of the Habib Bourguiba University Hospital (Sfax, Tunisia).

 For each specimen, the following pathological data were collected: tumor multifocality, tumor size, histological grade [according to Elston-Ellis modification of Scarff-Bloom-Richardson system (SBR)],^13^ presence of lymphovascular invasion (LVI), presence of perineural invasion (PNI), tumor necrosis, concomitant carcinoma *in situ* (CIS), Paget disease, surgical margin status, ER and PR status,^14^ HER2 status^15^ and proliferation index Ki-67 (considered overexpressed if ≥ 20%).^16^ HER2 staining was analyzed according to the Wolff criteria.^15^ Tumors were considered positive for HER2 if immunostaining was scored as 3 + . HER2 cases evaluated as 2 + were examined with fluorescent *in situ* hybridization (FISH). ER and PR were considered positive when more than 1% of the infiltrating tumor cell nuclei were marked.^14^

 We identified five molecular subtypes: Luminal A (LA) if ER and/or PR positive, HER2 negative and Ki-67 < 20%; Luminal B like (LB-Like) if ER and/or PR positive, HER2 negative and Ki- 67 > 20%; Luminal B (LB) if ER and/or PR positive and HER2 positive; HER2-positive breast cancer (HER2 +) if both ER and PR negative and HER2 positive and finally, Triple Negative Breast Cancer (TNBC) if HR and HER2 negative.^17^

 The pathological TNM classification and staging was done as per American Joint Committee on Cancer (AJCC) guidelines.^18^

 Clinical data were collected from medical records at the department of Oncology of the Habib Bourguiba University Hospital (Sfax, Tunisia) and they included age, menopausal status, distant metastasis at diagnosis and outcomes. Overall survival (OS) was designated as time from primary surgical treatment to the date of the last follow-up or death. Disease-free-survival (DFS) was designated as time during which no sign of cancer appeared after treatment.

###  Immunohistochemical Staining for Cyclin D1

 The expression of cyclin D1 (clone E1544; dilution 1:50; Spring) was evaluated by immunohistochemistry (IHC). We used 3 μm slices, dried overnight at 40°C and deparaffinized in xylene. Later, slices were rehydrated in alcohol at 100°C then at 95°C and washed in purified water.

 Antigen retrieval was evaluated using a boiled water bath with basic buffer (pH 9) for 40 minutes until the temperature climbed to 98°C. They were then allowed to cool spontaneously.

 The endogenous peroxidase activity was neutralized by hydrogen peroxide (H_2_O_2_, 3%) for 10 min. The sections were washed with distilled water and with phosphate buffered saline (PBS). The sections were then covered with antibody for cyclin D1 at 1:30 dilution for 1 hour. Next, sections were incubated with biotin-conjugated secondary antibody for 20 min and then incubated using streptavidin biotin system for 20 minutes at room temperature. PBS washing for 5 minutes was performed for each step. The reactions became clear after the immersion of sections in 3, 3 diaminobenzidine a substrate–chromogen solution for 20 minutes. The final step was to counterstain the slides with Mayer hematoxylin, to mount them permanently, and to examine them with a standard light microscope.

 Immunostaining for cyclin D1 was interpreted by two pathologists. It was considered positive when at least 10% or more of the tumor cells showed nuclear expression regardless of the intensity of staining.^2^

###  Statistical Analyses

 Data were analyzed using the SPSS software (version 20.0). The correlation between cyclin D1 expression and the clinical-pathological factors was evaluated by the Chi-square and the Fisher exact tests.

 Survival analysis was performed using the Kaplan-Meier method and compared by the log-rank test. A Cox regression model was used to describe the risk factors with survival parameters. A *P* value ≤ 0.05 showed statistical significance.

## Results

###  Population Characteristics

 Clinical and pathological data arelistedin Table 1. Paget disease was noted in 6 % of cases. Surgical margins were positive in 12 cases. The median follow-up was 53, 3 months. The 5-year OS rate was 82.1%. The 5-year DFS rate was 80.1%.

**Table 1:** Relationship between Cyclin D1 Expression and Clinical-Pathological Characteristics in Breast Cancer

**Variable**		**Cyclin D1 Expression**		
		**<10% (n=26)**	**≥10 (n=74)**	***P***
Age (y) (mean = 50.5 years)	≤ 45 (n = 35)	8 (22.9%)	27 (77.1%)	0.599
	> 45 (n = 65)	18 (27.7%)	47 (72.3%)	
Hormonal status	Menopausal (n = 45)	11 (24.5%)	34 (75.5%)	0.748
	Not menopausal (n = 55)	15 (27.3%)	40 (72.7%)	
Multifocality	Yes (n = 17)	2 (11.8%)	15 (88.2%)	0.225
	No (n = 83)	24 (29%)	59 (71%)	
Tumor size (cm)	≤ 2 (n = 23)	6 (73.9%)	17 (26.1%)	0.7
	2-5 (n = 62)	47 (75.8%)	15 (24.2%)	
	> 5 (n = 15)	5 (33.3%)	10 (66.7%)	
SBR Grading	Grade I (n = 16)	4 (25%)	12 (75%)	0.8
	Grade II (n = 61)	17 (27.9%)	44 (72.1%)	
	Grade III (n = 23)	5 (21.7%)	18 (78.3%)	
	Low Grade (Grade I,II) (n = 77)	21 (27.3%)	56 (72.7%)	**0.007**
	High Grade (Grade III) (n = 23)	5 (21.7%)	18 (78.3%)	
LVI	Yes (n = 56)	17 (30.3%)	39 (69.7%)	0.262
	No (n = 44)	9 (20.5%)	35 (79.5%)	
PNI	Yes (n = 27)	4 (14.8%)	23 (85.2%)	0.121
	No (n = 73)	22 (30.1%	51 (69.9%)	
Tumor necrosis	Yes (n = 18)	8 (44.4%)	10 (55.6%)	0.073
	No (n = 82)	18 (22%)	64 (78%)	
CIS	Yes (n = 81)	19 (23.5%)	62 (76.5%)	0.253
	No (n = 19)	7 (36.8%)	12 (63.2%)	
HER-2 expression status	Amplified (n = 28)	6 (21.4%)	22 (78.6%)	0.516
	Non amplified (n = 72)	20 (27.8%)	52 (72.2%)	
ER expression status	Positive (n = 74)	12 (16.2%)	62 (83.8%)	**<0.0001**
	Negative (n = 26)	14 (53.8%)	12 (46.2%)	
PR expression status	Positive (n = 63)	9 (14.3%)	54 (85.7%)	**<0.0001**
	Negative (n = 37)	17 (46%)	20 (54%)	
Ki67	≥ 20% (n = 50)	14 (28%)	36 (72%)	0.648
	< 20% (n = 50)	12 (24%)	38 (76%)	
Molecular subtype	LA (n = 20)	9 (45%)	11 (55%)	0.2
	LB- HER2 negative (n = 36)	8 (22.2%)	28 (77.8%)	
	LB-HER2 positive (n = 18)	5 (27.8%)	13 (72.2%)	
	HER2 + (n = 11)	2 (18.2%)	9 (81.8%)	
	TNBC (n = 15)	2 (86.7%)	13 (13.3%)	
pT status	p T1 (n = 23)	5 (21.7%)	18 (78.3%)	0.1
	p T2 (n = 54)	12 (22.2%)	42 (77.8%)	
	p T3 (n = 14)	4 (28.6%)	10 (71.4%)	
	p T4 (n = 9)	5 (55.6%)	4 (44.4%)	
Lymph node status	N0 (n = 42)	8 (19%)	34 (81%)	0.3
	N1 (n = 32)	12 (37.5%)	20 (62.5%)	
	N2 (n = 15)	4 (26.7%)	11 (73.3%)	
	N3 (n = 11)	2 (18.2%)	9 (81.8%)	
Metastasis	M + (n = 8)	1 (12.5%)	7 (87.5%)	0.676
	M – (n = 92)	25 (27.2%)	67 (72.8%)	

SBR, Scarff-Bloom-Richardson; LVI, Lymphovascular invasion; PNI, Perineural invasion; Her-2, Human Epidermal Growth 2; ER, estrogen receptor; PR, progesterone receptor; LA, luminal A; LB, Luminal B; TNBC, triple negative breast cancer Bold highlighted red values show significance (*P*≤ 0.05).

###  Cyclin D1 Expression and Relation with other Clinical-pathological Factors

**Figure 1:**
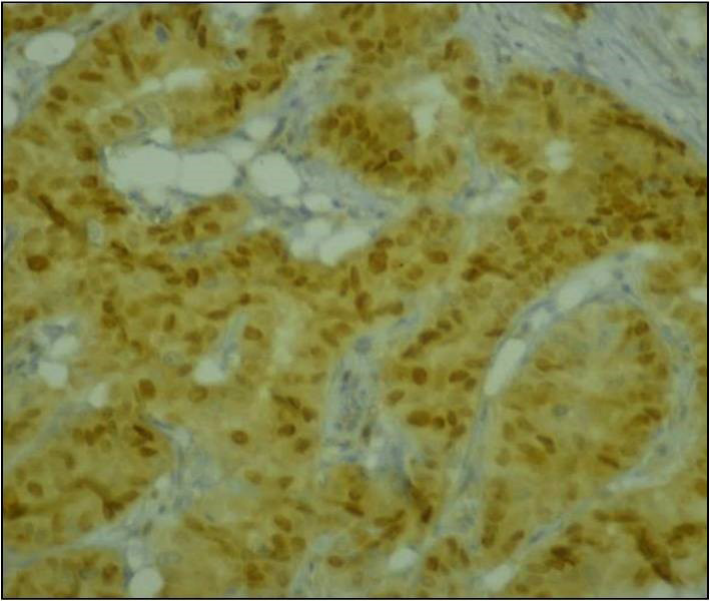
Cyclin D1 Immunostaining. Strong nuclear cyclin D1 staining (IHC × 200).

 Cyclin D1 was found to be positive in 74 cases, 32 of which showed strong immunoreactivity and 14 showed weak immunoreactivity (Figure 1).

 The relationbetween cyclin D1 expression and clinical-pathological factors is shown in Table 1. Cyclin D1 staining was significantly correlated with ER and PR positivity (*P*< 0.0001), with low grade SBR (*P* = 0.007).

 Most cases of positive cyclin D1 showed absence of tumor necrosis (*P*= 0.073). Moreover, none of the clinical data and other pathological features showed any association with cyclin D1 expression (Table 1).

**Figure 2:**
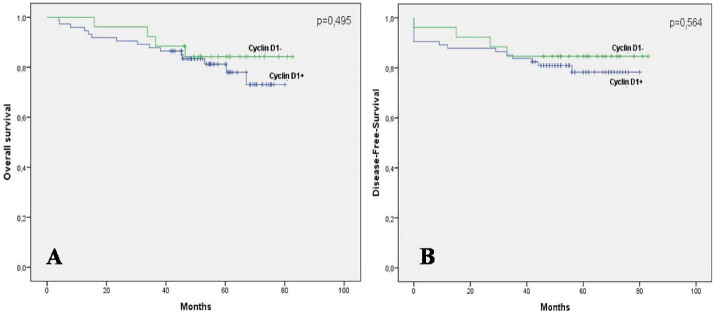
Survival Outcomes According to Cyclin D1 Expression. **(A)** Kaplan-Meier curves of overall survival according to Cyclin D1 expression: the 5-year OS rate was 81.2% in tumors with positive staining for Cyclin D1 and 84.2% in tumors without Cyclin D1 expression (p = 0.459).** (B) **Kaplan-Meier curves of Disease-Free-survival according to Cyclin D1 expression: the expression of Cyclin D1 was not associated with better DFS (*P* = 0.564).

 Univariate analysis showedthat expression of cyclin D1 was not statistically correlatedwith OS and DFS. The 5-year OS rate was 81.2% in tumors with positive staining for cyclin D1 and 84.2% in tumors without cyclin D1 expression (*P*= 0.459) (Figure 2A). Similarly, the expression of cyclin D1 was not associated with better DFS (*P*= 0.564) (Figure 2B).

 A multivariate Cox model was performed to evaluate the risk factorsassociated with OS and DFS. Only factors which were a significant prognostic factor in the univariate study were included in the model (Tumor size, SBR grade, LVI, pT status, pN status and metastasis). The results are presented in Table 2 and Table 3.

**Table 2:** Results of Multivariate Cox Regression Model for Overall Survival

**Variables**	**Coefficient**	**Errer** **Standard**	***P*** ** Value**	**HR**	**95% CI**	
					**Lower**	**Upper**
Tumor size	1.312	0.883	0.137	3.712	0.658	20.932
SBR Grade	1.514	0.672	**0.024**	4.546	1.219	16.956
LVI	12.668	388.107	0.974	1.52	0.000	0
pT status	0.014	0.484	0.978	1.014	0.393	2.616
pN status	0.459	0.389	0.238	1.583	0.739	3.391
Metastasis	4.418	1.252	**0.000**	82.909	7.127	964.494

HR, hazard ratio; 95% CI, 95% confidence interval; SBR, Scarff-Bloom-Richardson; LVI, lymphovascular invasion. Bold highlighted values show significance (*P* ≤ 0.05).

**Table 3:** Results of Multivariate Cox Regression Model for Disease Free Survival

**Variables**	**Coefficient**	**Errer Standard**	***P *** **Value**	**HR**	**95% CI**	
					**Lower**	**Upper**
Tumor size	0.824	0.775	0.288	2.281	0.499	10.423
SBR Grade	1.215	0.594	**0.041**	3.371	1.053	10.796
LVI	12.224	283.428	0.966	1.103	0.000	0
pT status	0.423	0.384	0.270	1.527	0.720	3.239
pN status	0.554	0.364	0.129	1.740	0.852	3.552

HR, hazard ratio; 95% CI, 95% confidence interval; SBR, Scarff-Bloom-Richardson; LVI, lymphovascular invasion. Bold highlighted values show significance (*P* ≤ 0.05).

## Discussion

###  Immunohistochemical Overexpression of Cyclin D1

 Overexpression of cyclin D1 varies from 23% to 81.4% according to studies published in the English literature.^19,20^ In our study, overexpression of cyclin D1 was noted in 74% of cases.

 This large difference between different series may be explained by several factors. Indeed, immunohistochemistry is performed using different clones of cyclin D1 which require specific protocol for each one and thus influence cyclin D1 staining. Next, this large discrepancy may also be influenced by the intrinsic characteristics of tumors.

 The overexpression of cyclin D1 may be detected by immunohistochemistry, using antibody against cyclin D1, even without any apparent increase in the copy numbers of CCND1.^21^

###  Association of Cyclin D1 Expression with Clinical-Pathological Data

 The majority of studies have shown a significant association between overexpression of cyclin D1 and good prognostic parameters such as small tumor size and good tumor differentiation with low grade SBR.^7,22,23^ A significant correlation between expression of cyclin D1 and low tumor grade has been reported by many authors, which is in line with our results.^23-25^

 Indeed, cyclin D1 affects cell maturation and differentiation; high expression of cyclin D1 suppresses deoxyribonucleic acid (DNA) replication by linking to the proliferating cell nuclear antigen and to cyclin-dependent kinase 2 (CDK2). ^6^

 A significant association has been reported in almost all publications between cyclin D1 overexpression and breast cancer subtypes ER-positive.^2,7,8,22,26-31^

 The expression of cyclin D1 was significantly correlated with PR and ER positivity in the present study (*P* < 0.0001).^2,9,23,32^

 In a study by Huang *et al.* on 101 cases of breast invasive carcinoma of no specific type, the overexpression of cyclin D1 was statistically correlated with early tumor stage (Stage I or II) (*P* = 0.047); 85% of these tumors were positive for ER (*P* < 0.0001) and 78.75% were positive for PR (*P* = 0.001).^7^

 Likewise, Mylona et al demonstrated that overexpression of cyclin D1 was associated with small tumors (*P* = 0.017), ER + (*P* < 0.0001), RP + (*P* < 0.0001), low grade SBR (*P* < 0.0001) and with low expression of protein 53 (p53) (*P* < 0.001).^33^

 In fact, estrogen and progesterone increase the transcription of the *CCND1* gene, thus leading to an overexpression of the cyclin D1 protein.^34^ So, Luminal A or B tumors express more cyclin D1 whereas tumors with basal-like phenotype lose this expression.^7,31^

 Studies showed that cyclin D1 acts like a transcriptional factor without any interaction with its associated CDKs.^35^ Somehow, it can bind to the hormone binding domain of the ER, thus activating the mediators and the transcriptional regulators of the ER.^10,36^ Simultaneously, this estrogen-cyclin D1 linkage acts by enhancing the PR expression through a novel estrogen- and cyclin D1-responsive enhancer in the PR gene.^35^ Consequently, cyclin D1 has a pivotal role in augmenting estrogen and progesterone effects in the mammary gland.^35^

 This CDK-independent cyclin D1 function leads to poor response to anti-estrogen treatment by acquiring an agonist effect.^36^ In fact, Kenny et al^37^ and Stendahl et al^38^ reported that overexpression of cyclin D1 is a predictor of resistance to hormonotherapy based on tamoxifen.

 Its prognostic value in terms of survival has been a subject of controversy; indeed, many authors have shown that cyclin D1 overexpression in breast cancer is correlated with a good outcome like Chung *et al.* who found that the 5-year-overall survival increased from 89.9% for cyclin D1 overexpression tumors to 98.9% for cyclin D1 negative tumors (*P*= 0.008).^20^ In this same study, the overexpression of cyclin D1 was correlated with a longer survival rate after tumor recurrence – 61 months for tumor overexpressing cyclin D1 versus 26 months for tumor with negative cyclin D1 (*P*= 0.012).^20^ Indeed, some authors reported that overexpression of cyclin D1 showed a statistically significant association with low proliferative rate.^24,25^ Thus, cyclin D1 overexpression could be a marker of a less indolent progression of the disease after tumor recurrence.^20^

 On the other hand, some authors have not shown a significant association in terms of OS or DFS which is consistent with our results.^25,28,39^

 However, some authors have reported that deletion of cyclin D1 can play a protector role, thus protecting against the development of breast carcinoma.^10,40^ In fact, cyclin D1 overexpression can be a predictor of poor prognosis.^37,41,42^ Ahlin et al found that cyclin D1 overexpression was associated with a high proliferative index and an increased risk of mortality in patients with ER positive breast cancer but not in those with ER negative breast cancer.^43^

 Given these findings, the relationship between cyclin D1 overexpression and hormone receptors in breast cancer seems to be well established among authors. Researches about new prognostic markers in breast cancer are currently in progress and provide a huge amount of information in terms of better understanding the tumor microenvironment^44^ and the gene signature.^45^ However, the prognostic value of cyclin D1 overexpression in breast cancer is controversial and needs further investigations.

 Actually, cyclin D1 is arising as an important prognostic parameter in invasive breast cancer, but it is not employed yet in routine practice. Its substantial role in predicting treatment response to tamoxifen should be considered to improve the management of ER-positive tumors.

 It constitutes a novel therapeutic target especially in patients with ER positive tumors to overcome resistance to tamoxifen.
